# Molecular Cloning and Characterization of Different Expression of *MYOZ2* and *MYOZ3* in Tianfu Goat

**DOI:** 10.1371/journal.pone.0082550

**Published:** 2013-12-18

**Authors:** Lu Wan, Jisi Ma, Nianlu Wang, Daihua Wang, Gangyi Xu

**Affiliations:** 1 Institute of Animal Genetics and Breeding, College of Animal Science and Technology, Sichuan Agricultural University, Ya’an, Sichuan, China; 2 Mianyang Agriculture Bureau, Mianyang, Sichuan, China; University of South Florida College of Medicine, United States of America

## Abstract

The myozenin family of proteins binds calcineurin, which is involved in myocyte differentiation of skeletal muscle. Moreover, gene expression of myozenin is closely related to meat quality. To further understand the functions and effects of myozenin2 (*MYOZ2*) and myozenin3 (*MYOZ3*) genes in goat, we cloned them from Tianfu goat longissimus dorsi muscle. Sequence analyses revealed that full-length coding sequence of *MYOZ2* consisted of 795 bp and encoded 264 amino acids, and full-length coding sequence of *MYOZ3* consisted of 735 bp and encoded 244 amino acids. RT-qPCR analyses revealed that mRNA expressions of *MYOZ2* and *MYOZ3* were detected in heart, liver, spleen, lung, kidney, leg muscle, abdominal muscle, and longissimus dorsi muscle. Particularly high expression levels of *MYOZ2* were seen in abdominal muscle and heart (*P*<0.01), low expression levels were seen in leg muscle (*P*<0.01), longissimus dorsi muscle (*P*>0.05) and very little expression were detected in liver, spleen, lung and kidney (*P*>0.05). In addition, high expression levels of *MYOZ3* were seen in abdominal muscle, leg muscle, lungs and kidney (*P*<0.01), low expression levels were found in longissimus dorsi muscle and spleen (*P*<0.01) and very little expression were detected in heart and liver (*P*>0.05). Temporal mRNA expression results showed that *MYOZ2* and *MYOZ3* gene expression varied across four muscle tissues with different ages of the goats. Western blotting further revealed that MYOZ2 and MYOZ3 proteins were only expressed in goat muscle, with notable temporal expression differences in specialized muscle tissues from five development age stages. This work provides the first evidence that *MYOZ2* and *MYOZ3* genes are expressed abundantly in Tianfu goat muscle tissues from different development age stages, and lay a foundation for understanding the functions of *MYOZ2* and *MYOZ3* genes in muscle fiber differentiation.

## Introduction

The quality of meat has become an important research area in recent years, and has many influencing factors. These factors include muscle fibers [1], [2], which are the basic unit of muscle and can be divided into four types: slow-oxidative (Type I), fast oxido-glycolytic (Type IIA), and fast glycolytic (Types IIX and IIB) [3]–[5]. The best quality meat has a high muscle content of mainly slow-twitch muscle fibers [6].

Calcineurin (CaN), a Ca^2+^/calmodulin dependent protein serine/threonine phosphatase is broadly distributed in various mammalian cells, where it is involved in the regulation of cellular function [7]. In skeletal muscle, it is required for the key processes of myocyte differentiation and conversion to the slow (oxidative) muscle phenotype [8]–[11].

Myozenin is an α-actinin- and γ-filamin-binding protein of skeletal muscle Z lines [12], and also is a family of muscle proteins that bind to calcineurin [13]. In other reports, the myozenin family termed it calsarcin [12], FATZ [14] and c4orf5 [15]. The expressions of myozenin genes, including myozenin1 (*MYOZ1*), myozenin2 (*MYOZ2*), and myozenin3 (*MYOZ3*), are closely related to meat quality. For example, the expression of *MYOZ2* is restricted to slow-twitch skeletal muscle and heart, while that of both *MYOZ1* and *MYOZ3* are enriched in fast-twitch skeletal muscle in adult animals [13], [16], [17]. In addition, the expression of *MYOZ2* effectively inhibits calcineurin activity, thereby regulating the differentiation of muscle fibers [18].

Many studies have been carried out into the role of *MYOZ2* in cardiac hypertrophy [19]–[21]. However, although *MYOZ2* and *MYOZ3* genes have been isolated and their expression been analyzed in other mammals, few such reports have been carried out in goats. The Tianfu goat is an emerging breed in China, and is famous for its good meat quality [22]. Here, we cloned Tianfu goat *MYOZ2* and *MYOZ3* genes, analyzed their cDNA and encored protein sequences, and determined their spatio-temporal expressions in different tissues by RT-qPCR and western blotting. This provides valuable information for the application of *MYOZ2* and *MYOZ3* in goat meat quality.

## Materials and Methods

### Experimental animals and sample collection

This study was carried out in strict accordance with the recommendations in the Guide for Sichuan Agricultural University Animal Care and Use Committee, Sichuan Agricultural University, Sichuan, China under permit no. DKY-B20100805, and all efforts were made to minimize suffering. All Tianfu goats were bred under standard conditions and slaughtered on day 1, day 75, day 150, day 225, and day 300 of life (n = 5 per group). The autopsy samples, including heart, cardiac muscle, liver, spleen, lung, kidney, leg muscle, abdominal muscle, longissimus dorsi muscle and soleus muscle samples were immediately dissected from each goat after slaughtering, stored at –80°C and detected for total RNA and protein extraction.

### Cloning of Tianfu goat *MYOZ2* and *MYOZ3* genes

To verify and clone the cDNA sequences of Tianfu goat *MYOZ2* and *MYOZ3*, total RNA was extracted from Tianfu goat longissimus dorsi muscle using RNAiso Plus (TaKaRa, Dalian, China). First strand cDNA was synthesised from total RNA using the PrimeScript™ RT reagent kit (TaKaRa) according to the manufacturer’s instructions. The primers (Table S1) for Tianfu goat *MYOZ2* and *MYOZ3* genes were designed from sequences of sheep *MYOZ2* and *MYOZ3* genes (*MYOZ2*: NM_001199222.1, *MYOZ3*: NM_001199795.1). PCRs were set up using 10-µl volumes containing 5 µl Taq RCR Mix (TaKaRa), 1 µl cDNA of Tianfu goat longissimus dorsi muscle, 0.5 µl PCR Forward primer, 0.5 µl PCR Reverse primer and 3 µl RNase-free H_2_O (Tiangen, Beijing, China). PCRs were run under the following cycling conditions: 95°C for 5 min, followed by 36 cycles of 95°C for 30 s, Tm (55°C and 56.3°C, respectively) for 30 s, 72°C for 1 min, and a final extension of 10 min at 72°C. PCR products were detected by 1.5% agarose gel electrophoresis, and recovered using an E.Z.N.A Gel Extraction Kit (Omega Bio-Tek, Winooski, VT, America). The products of gel extraction purification were cloned into pMD19-T vector (TaKaRa) according to standard protocols and sequenced by LiuHe HuaDa Biotechnology (Beijing) Co., Ltd. (Beijing, China).

### Analyzing sequences of *MYOZ2* and *MYOZ3* genes

ORF Finder (http://www.ncbi.nlm.nih.gov/gorf/orfig.cgi) was used to identify open reading frames (ORFs), and sequence analysis of Tianfu goat *MYOZ2* and *MYOZ3* genes were performed using online software NCBI (http://www.ncbi.nlm.nih.gov) and ExPaSy (http://www.expasy.org). The NetPhos 2.0 server (http://www.cbs.dtu.dk/services/NetPhos/) was used to produce neural network predictions for serine, threonine and tyrosine phosphorylation sites in eukaryotic proteins, while the SignaIP 4.1 server (http://www.cbs.dtu.dk/services/SignalP/) was determined the location of signal peptide cleavage sites in amino acid sequences. Transmembrane helices were predicted using TMHMM 2.0 (http://www.cbs.dtu.dk/services/TMHMM-2.0/). The coding nucleotide and protein sequences of *MYOZ2* and *MYOZ3* from related different species were aligned with the goats sequence using the DNAMAN V6 software (Lynnon Biosoft, America). The secondary structures of protein were predicted by NPS@ (http://npsa-pbil.ibcp.fr/cgi-bin/npsa_automat.pl?page=/NPSA/%20npsa_hnn.html\). The Batch Web CD-search (http://www.ncbi.nlm.nih.gov/Structure/bwrpsb/bwrpsb.cgi) and the SMART (http://smart.embl-heidelberg.de/smart/set_mode.cgi?NORMAL=1) were used to predict the putative conserved protein domains. The amino acid sequences of MYOZ2 and MYOZ3 used for construction of the phylogenic tree were completed using the MEGA 5.10 program.

### RT-qPCR analysis of *MYOZ2* and *MYOZ3* expression

Temporal and spatial mRNA expression patterns of Tianfu goat *MYOZ2* and *MYOZ3* genes were analyzed by RT-qPCR using the constitutively expressed Glyceraldeyhyde-3-phosphate dehydrogenase (*GAPDH*) gene as an internal control with the primers shown in Table S1. Total RNAs were extracted from different tissues using RNAiso Plus (TaKaRa), and treated with RNase-free H_2_O (Tiangen) according to the manufacturer's instructions. Spatial mRNA expression analysis samples were harvested on day 300, and included heart, liver, spleen, lung, kidney, leg muscle, abdominal muscle, and longissimus dorsi muscle (n = 5). Temporal mRNA expression analysis samples were harvested on days 1, 75, 150, 225, and 300, and included cardiac muscle, leg muscle, abdominal muscle, and longissimus dorsi muscle (n = 5). The 25 µl reaction system included 12.5 µl SYBR premix Ex TAq™ (TaKaRa), 1 µl RT-qPCR Forward primer, 1 µl RT-qPCR Reverse primer, 2 µl cDNA of each tissue, and 8.5 µl RNase-free H_2_O (Tiangen). PCR amplification was carried out as follows: 95°C for 10 s followed by 40 cycles of 95°C for 5 s and 60°C for 30 s. Each sample was repeated three times. The 2^–ΔΔCt^ method was used to analyze the mRNA expression levels of *MYOZ2* and *MYOZ3* [23].

### Western blotting analyses of MYOZ2 and MYOZ3 protein expression

Western blotting was used to analyze the relative protein expression of MYOZ2 and MYOZ3 proteins in Tianfu goats. Samples for spatial protein expression were harvested on day 300, and included heart, liver, spleen, lung, kidney, leg muscle, abdominal muscle, and longissimus dorsi muscle (n = 5). Samples for temporal protein expression were harvested on days 1, 75, 150, 225, and 300, and included longissimus dorsi muscle and soleus muscle (n = 5). Total proteins were extracted according to the laboratory established methods [24] to standard procedures and concentrations were determined in pre-cleared extracts using the BCA protein assay kit (BiYunTian, Shanghai, China) with BSA as a standard. The GAPDH protein was selected as the internal control. Polyclonal antibodies to MYOZ2 and MYOZ3 proteins (Abcam, England) were used at 1/500 dilutions, while a monoclonal antibody to GAPDH (BiYunTian) was used at a dilution of 1/1,000. The extracts were run on 12% SDS-polyacrylamide gels (12 µg total proteins per lane). As detailed previously [24], densitometric analysis was used to estimate the relative protein expression levels of MYOZ2 and MYOZ3.

### Statistical analysis

All data are expressed as means ± SEM. Statistical analysis was performed using one-way ANOVA with the SPSS 10.0 statistical software package (SPSS Inc., Chicago, IL, USA). The threshold of significance was defined as *P*<0.05.

## Results

### Nucleotide and protein sequences analysis

The nucleotide sequences analysis revealed that Tianfu goat *MYOZ2* and *MYOZ3* genes were not homologous to any known caprine genes, while a BLAST search revealed that they were significantly similar to the *MYOZ2* and *MYOZ3* gene sequences of other mammals. Full-length Tianfu goat *MYOZ2* cDNA was shown to be 806 bp in length and to contain an ORF of 795 bp encoding a protein of 264 residues with an expected molecular weight of 30.0 kDa and an isoelectric point (pI) of 6.99. Full-length Tianfu goat *MYOZ3* cDNA was 827 bp long with an ORF of 735 bp encoding a protein of 244 residues with an estimated molecular weight of 26.8 kDa and an isoelectric point (pI) of 8.94. Tianfu goat *MYOZ2* and *MYOZ3* gene sequences were deposited in GenBank (GenBank: JX573191 and KC537058, respectively). The coding nucleotide sequences of *MYOZ2* were 99.50%, 98.49%, 94.97%, and 91.82% identical to ovine, bovine, porcine, and human sequences, respectively, while those of *MYOZ3* were 98.50%, 96.61%, 84.55%, and 82.80% identical to ovine, bovine, porcine, and human sequences, respectively.

Analysis of the deduced amino acid sequences of Tianfu goat MYOZ2 and MYOZ3 proteins revealed the existence of several potential phosphorylation sites (19 in MYOZ2 and 15 in MYOZ3) but no signal peptides or transmembrane regions. The secondary structures of both proteins were predicted to be mainly comprised of α-helices and random coils. The Batch Web CD-search and SMART online software identified that a calsarcin domain was conserved in both proteins (amino acids 1–264 in MYOZ2 and amino acids 1–244 in MYOZ3). This domain consists of calcineurin-binding proteins, and has been implicated in the transduction of signals that control cardiac muscle hypertrophy and gene expression of slow fiber in skeletal muscle.

Sequence alignment using DNAMAN V6 software revealed that the encoded amino acid sequence of Tianfu goat MYOZ2 was 100% identical to ovine MYOZ2, and 98.48%, 95.08%, and 90.15% identical to that of bovine, porcine, and human, respectively. The encoded amino acid sequence of Tianfu goat MYOZ3 was 97.13% identical to ovine MYOZ3, and 95.92%, 82.04%, and 79.68% identical to that of bovine, porcine, and human, respectively. Protein sequence alignment of Tianfu goat MYOZ2 and MYOZ3 is shown in Figure S1.

Phylogenetic tree analysis was applied to determine the phylogenetic positions of Tianfu goat MYOZ2 and MYOZ3 in relation to that of 11 different species. All MYOZ2 and MYOZ3 members in the listed species could be placed into two distinct groups. The highest homology of Tianfu goat MYOZ2 and MYOZ3 was with that of sheep, and the lowest was with that of African clawed frog. Moreover, MYOZ2 was found to have diverged earlier than MYOZ3 (Figure S2).

### Temporal and spatial mRNA expression patterns

RT-qPCR analyses revealed that the expressions of *MYOZ2* and *MYOZ3* genes were detected in the heart, liver, spleen, lung, kidney, leg muscle, abdominal muscle, and longissimus dorsi muscle on day 300. Particularly high expression levels of *MYOZ2* were found in abdominal muscle and heart (*P*<0.01), while lower levels were found in leg muscle (*P*<0.01) and longissimus dorsi muscle (*P*>0.05); very little expression was detected in liver, spleen, lung, and kidney (*P*>0.05) (Figure S3). High expression levels of *MYOZ3* were seen in abdominal muscle, leg muscle, lung, and kidney (*P*<0.01), while lower levels were detected in longissimus dorsi muscle and the spleen (*P*<0.01); very little expression was detected in heart and liver (*P*>0.05) (Figure S3).

Temporal mRNA expressions of both *MYOZ2* and *MYOZ3* genes were found to follow a trend according to postnatal age of Tianfu goat (Figure S4 and Figure S5). During cardiac muscle development, gene expression of *MYOZ2* was first increased from day 1 to day 150, then gradually decreased to day 300; the highest expression was seen on day 150 and the lowest expression on day 300 (Figure S4). In leg muscle, gene expression of *MYOZ2* was first increased from day 1 to day 75, then decreased to day 150, after which it increased to day 225, and last decreased to day 300; the highest expression was seen on day 75 and the lowest on day 300 (Figure S4). This contrasts with expression in abdominal muscle, which was highest on day 1 and lowest on day 75 (Figure S4). In longissimus dorsi muscle, gene expression of *MYOZ2* was gradually decreased from day 1 to day 225, after which it increased to day 300; the highest expression was observed on day 1 and the lowest on day 225 (Figure S4).

Gene expression of *MYOZ3* was decreased in cardiac muscle, leg muscle, and abdominal muscle but increased in longissimus dorsi muscle from day 1 to day 75; it then was increased in all these muscle tissues from day 75 to day 150 (Figure S5). From day 150 to day 225, gene expression of *MYOZ3* was increased in cardiac muscle but decreased in leg muscle, abdominal muscle, and longissimus dorsi muscle (Figure S5). From day 225 to day 300, gene expression of *MYOZ3* was decreased in cardiac muscle but increased in leg muscle, abdominal muscle, and longissimus dorsi muscle (Figure S5). During cardiac muscle development, the highest expression level occurred on day 1 and the lowest was on day 300; in leg muscle, these levels were seen on days 150 and 75, respectively; in abdominal muscle, on days 1 and 225, respectively; and in longissimus dorsi muscle, on days 150 and 1, respectively (Figure S5).

### Temporal and spatial protein expression patterns

Western blotting revealed that different levels of MYOZ2 and MYOZ3 proteins were expressed in the heart, liver, spleen, lung, kidney, leg muscle, abdominal muscle, and longissimus dorsi muscle on day 300. Using GAPDH as the reference protein, no MYOZ2 and MYOZ3 was detected in liver, spleen, lung, and kidney. However, both proteins were highly expressed in abdominal muscle and leg muscle, while lower levels were detected in different tissues. The lowest expression level of MYOZ2 was in longissimus dorsi muscle, and that of MYOZ3 was found in heart (Figure S6).

Temporal protein expression results showed different levels of MYOZ2 and MYOZ3 proteins in longissimus dorsi muscle and soleus muscle from day 1 to day 300. Different protein expression trends were seen in specialized muscle tissues. Thus, the protein expression of MYOZ2 was reduced in longissimus dorsi muscle with increasing age of the Tianfu goats: high expression levels were found on days 1, 75, and 150, while low levels were seen on days 225 and 300 (Figure S7). Protein expression of MYOZ3 was first increased then decreased in longissimus dorsi muscle with increasing goat age; highest expression levels were found on days 75 and 150, while low expression levels were seen on days 1, 225, and 300 (Figure S7). Moreover, during soleus muscle growth and development, an upward trend of MYOZ2 expression was seen, with high levels on days 150, 225, and 300, and low levels on days 1 and 75 (Figure S8). In the same muscle, protein expression of MYOZ3 was first increased from day 1 to 75, then decreased from day 75 to 300; highest expression levels were seen on days 75 and 150, and lowest levels on days 1, 225, and 300 (Figure S8).

## Discussion

With the improvement of living standards, people have an increasingly higher demand for the consumption of meat. The quality of meat is one of the most important economic traits in domestic animals, and is determined at least in part by muscle fibers, which are under the control of multiple gene products [25]–[27]. In cardiac and skeletal muscle, the products of *MYOZ2* and *MYOZ3* genes appear to influence the expression of calcineurin, which plays an important role in hypertrophic cardiomyopathy and skeletal muscle fiber differentiation [13], [16]. Therefore, gene expression of *MYOZ2* and *MYOZ3* can directly affect meat quality. They are highly expressed in muscle tissues, suggesting that they are related to muscle growth and development. Moreover, recent studies in mice showed that gene expression of *MYOZ1* [28] and *MYOZ2* [18] can significantly decrease the expression of *calcineurin* gene [7].

### Characteristics of Tianfu goat *MYOZ2* and *MYOZ3* sequences

In our study, we cloned the cDNA sequences of Tianfu goat *MYOZ2* and *MYOZ3* genes, and analyzed their nucleotide and protein sequences. The 806 bp cDNA of Tianfu goat *MYOZ2* contained a 795 bp long ORF that was translated to produce a 264 amino acid sequence with a molecular mass of 30.0 kDa, and the 827 bp cDNA of Tianfu goat *MYOZ3*contained a 735 bp long ORF that was translated to produce a 244 amino acid sequence with a molecular mass of 26.8 kDa. To further understand structures and functions of Tianfu goat MYOZ2 and MYOZ3 proteins, their deduced amino acid sequences were predicted by bioinformatic analysis software. We found several similarities between the two protein sequences, including no signal peptides and transmembrane regions, constituent part of their secondary structures and the presence of a conserved calsarcin domain. This represents a novel family of sarcomeric proteins that link calcineurin with the contractile apparatus, thereby potentially coupling muscle activity to calcineurin activation [13], [16]. These findings suggest that Tianfu goat MYOZ2 and MYOZ3 proteins bind calcineurin.

Coding nucleotide and protein sequences alignment showed that *MYOZ2* was more highly conserved in large mammals than *MYZO3*. This is consistent with previous research in pigs [17], inferring that *MYOZ2* plays a more important role than *MYOZ3* in calcineurin activation. Protein sequence alignment of Tianfu goat MYOZ2 and MYZO3 showed their homologies areas (Figure S1), suggesting that their homologies areas may be functional areas, which may be the putative calcineurin-binding regions [13], [16]. In addition, the phylogenetic tree showed that the Tianfu goat *MYOZ2* gene had closer genetic relationships with other mammals [17], further suggesting that *MYOZ2* may be evolutionarily conserved and may play an important role in the basic metabolic functions (Figure S2).

### Different mRNA expression patterns of Tianfu goat *MYOZ2* and *MYOZ3*

The mRNA expressions of Tianfu goat *MYOZ2* and *MYOZ3* genes were shown similar tissue distributions, with highest levels seen in abdominal muscle and lowest in liver (Figure S3). In previous studies, both *MYOZ2* and *MYOZ3* genes were highly expressed in human [15], mouse [13], [16] and pig [17] muscle. Moreover, we previously found that an experience of our own confirmed that *MYOZ2* and *MYOZ3* genes were highly expressed in Tianfu goat muscle, suggesting that *MYOZ2* and *MYOZ3* genes may play a part in goat muscle (Figure S3). In the present study, Tianfu goat *MYOZ2* was enriched in heart compared with *MYOZ3*, suggesting that *MYOZ2* has a particular function in cardiac muscle [20], [21]. Indeed, based on the different expression patterns of *MYOZ2* and *MYOZ3* in human, mouse, pig, and goat muscle, we hypothesize that *MYOZ2* and *MYOZ3* play different roles in the development of different muscles.

Examination of different muscle tissues over time revealed differences in Tianfu goat *MYOZ2* and *MYOZ3* expression. Both Tianfu goat *MYOZ2* and *MYOZ3* genes were changed differently in four muscle tissues from five development stages (Figure S4 and Figure S5). After birth, muscle growth was mainly to be muscle fiber hypertrophy and muscle fiber differentiation, and muscle fiber type transitions did not proceed immediately changes in mammal [5], [29], [30]. During this time, Tianfu goat *MYOZ2* and *MYOZ3* genes showed varied expression patterns in different muscle tissues, implying that they have different roles in muscle fiber differentiation (Figure S4 and Figure S5). Thus, in goats, the biological activities associated with *MYOZ2* and *MYOZ3* gene functions may differ according to tissue and development stage, confirming that they are relevant candidate genes for the control of meat quality.

### Different protein expression patterns of Tianfu goat MYOZ2 and MYOZ3

Protein is an embodiment of those life activities, and its expression has important biological significance. In this work, MYOZ2 and MYOZ3 were shown to be muscle-specific proteins in Tianfu goats (Figure S6), which is to be expected since myozenin is an α-actinin and γ-filamin-binding protein of skeletal muscle Z lines [12], [14]. In the present study, we further detected protein expressions of Tianfu goat MYOZ2 and MYOZ3 in longissimus dorsi muscle and soleus muscle from five age stages. Tianfu goat MYOZ2 and MYOZ3 proteins showed different expression trends in two specialized muscle tissues. In other fast and slow muscles studies, during postnatal development, longissimus dorsi muscle was a fast white muscle [31], [32] and soleus muscle was a slow red muscle [33], [34]. During that five age stages, this may represent the differentiation of fast and slow muscle fibers. In virtue of fast and slow muscle fibers differentiation, the MYOZ2 and MYOZ3 protein expressions were showed different variation trend in two muscle tissues (Figure S7 and Figure S8). We also found that MYOZ2 was enriched in slow-twitch fibers of goat, which supports a previous finding in pigs [17]. Due to different protein expression patterns of MYOZ2 and MYOZ3 in different muscle fiber from five age stages, these results further implied that *MYOZ2* and *MYOZ3* may serve a different function in controlling muscle fibers type.

In summary, we have isolated Tianfu goat *MYOZ2* and *MYOZ3* genes for the first time, performed sequences analysis and analyzed temporal and spatial expression differences using RT-qPCR and western blotting in Tianfu goat. These data presented here lay the foundations for future research on the functions of *MYOZ2* and *MYOZ3* genes in muscle fiber differentiation.

## Supporting Information

Figure S1 **Protein sequence alignment of Tianfu goat MYOZ2 and MYZO3.** Conserved amino acids are highlighted. The putative calcineurin-binding regions are underlined.
(TIF)

Figure S2 **The phylogenetic tree of MYOZ2 and MYZO3.** Note: Sequences shown are from NCBI sequence database. Sequences of MYOZ2 and MYZO3 are referred to in Table S2.
(TIF)

Figure S3 **Spatial mRNA expression profile of Tianfu goat** ***MYOZ2*** **and** ***MYOZ3*** **genes.** Note: The samples 1-8 represent heart, liver, spleen, lung, kidney, leg muscle, abdomial muscle and longissimus dorsi muscle, respectively. Bars represent the mean ± SE (n = 5).
(TIF)

Figure S4 **Temporal mRNA expression profiles of Tianfu goat** ***MYOZ2*** **gene during different muscle development.** Note: The samples 1-5 represent the 1st day, 75th day, 150th day, 225th day and 300th day, respectively; the samples a-d represent cardiac muscle, leg muscle, abdomial muscle and longissimus dorsi muscle, respectively. Bars represent the mean ± SE (n = 5).
(TIF)

Figure S5 **Temporal mRNA expression profiles of Tianfu goat** ***MYOZ3*** **gene during different muscle development.** Note: The samples 1-5 represent the 1th day, 75th day, 150th day, 225th day and 300th day, respectively; the samples a-d represent cardiac muscle, leg muscle, abdomial muscle and longissimus dorsi muscle, respectively. Bars represent the mean ± SE (n = 5).
(TIF)

Figure S6 **Western blotting of Tianfu goat MYOZ2 and MYOZ3 protein levels in eight tissues.** Note: The samples 1-8 represent heart, liver, spleen, lung, kidney, leg muscle, abdomial muscle and longissimus dorsi muscle, respectively (n = 5).
(TIF)

Figure S7 **Western blotting of Tianfu goat MYOZ2 and MYOZ3 protein levels during longissimus dorsi muscle development.** Note: The samples 1-5 represent the 1st day, 75th day, 150th day, 225th day and 300th day, respectively (n = 5).
(TIF)

Figure S8 **Western blotting of Tianfu goat MYOZ2 and MYOZ3 protein levels during soleus muscle development.** Note: The samples 1-5 represent the 1st day, 75th day, 150th day, 225th day and 300th day, respectively (n = 5).
(TIF)

Table S1 **Primers used for analysis of the expression of MYOZ2 and MYOZ3 genes.**
(XLS)

Table S2 **List of the MYOZ2 and MYOZ3 sequences used in phylogenetic tree analysis**.
(XLS)
