# Supplementary figures and images for: Molecular Cloning and Characterization of Different Expression of MYOZ2 and MYOZ3 in Tianfu Goat
Source: PLoS One. 2013 Dec 18;8(12):e82550. doi: 10.1371/journal.pone.0082550 (PMC3867352; doi:10.1371/journal.pone.0082550)

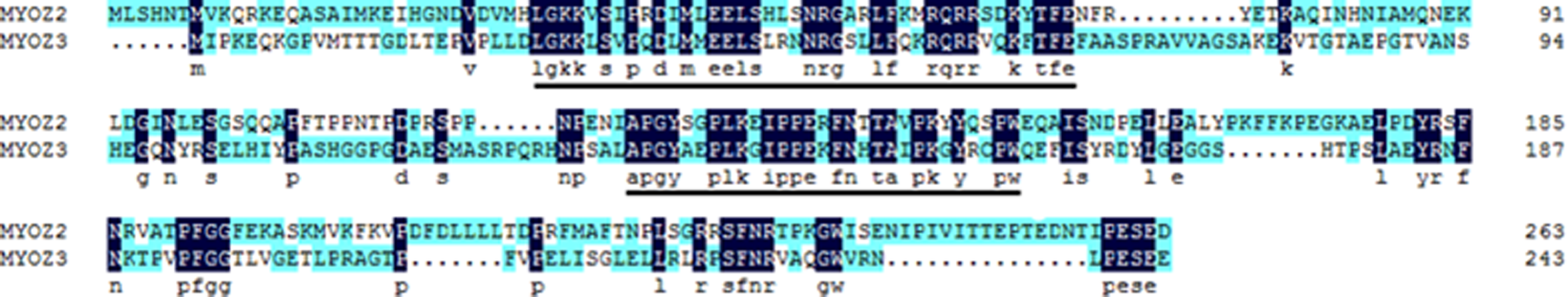

Supplement: Figure S1 — Protein sequence alignment of Tianfu goat MYOZ2 and MYZO3. Conserved amino acids are highlighted. The putative calcineurin-binding regions are underlined. (TIF) [file pone.0082550.s001.tif]

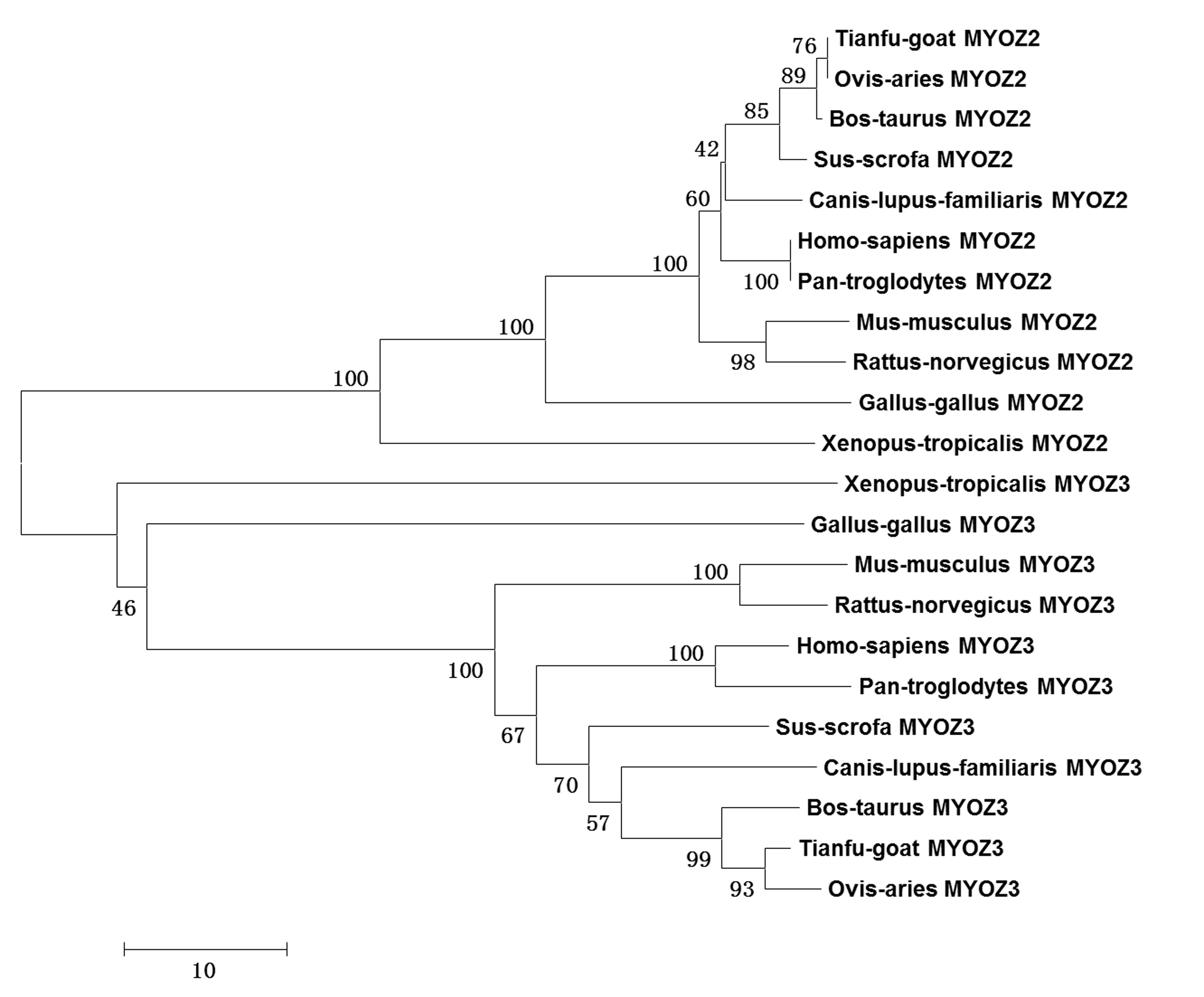

Supplement: Figure S2 — The phylogenetic tree of MYOZ2 and MYZO3. Note: Sequences shown are from NCBI sequence database. Sequences of MYOZ2 and MYZO3 are referred to in Table S2. (TIF) [file pone.0082550.s002.tif]

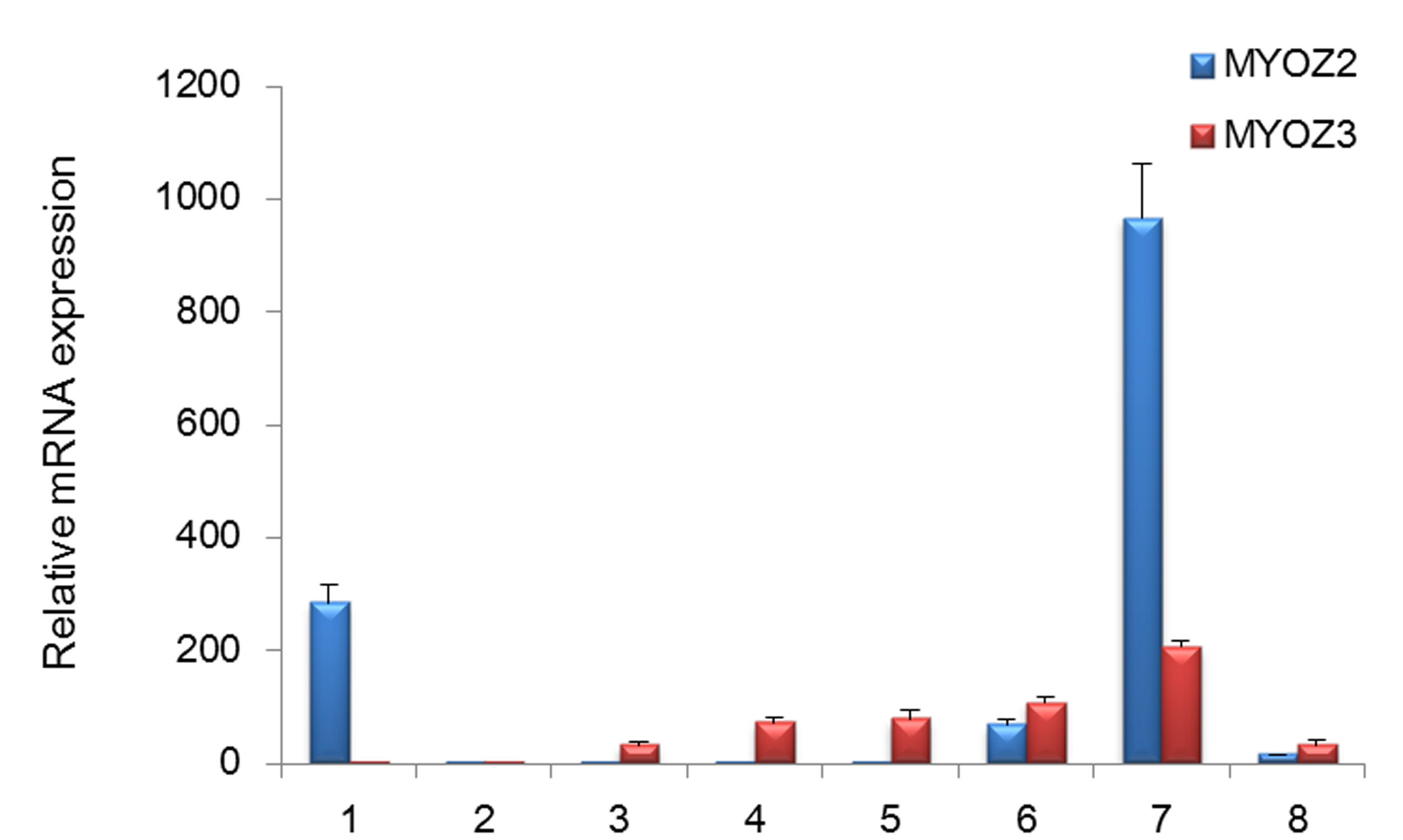

Supplement: Figure S3 — Spatial mRNA expression profile of Tianfu goat MYOZ2 and MYOZ3 genes. Note: The samples 1-8 represent heart, liver, spleen, lung, kidney, leg muscle, abdomial muscle and longissimus dorsi muscle, respectively. Bars represent the mean ± SE (n = 5). (TIF) [file pone.0082550.s003.tif]

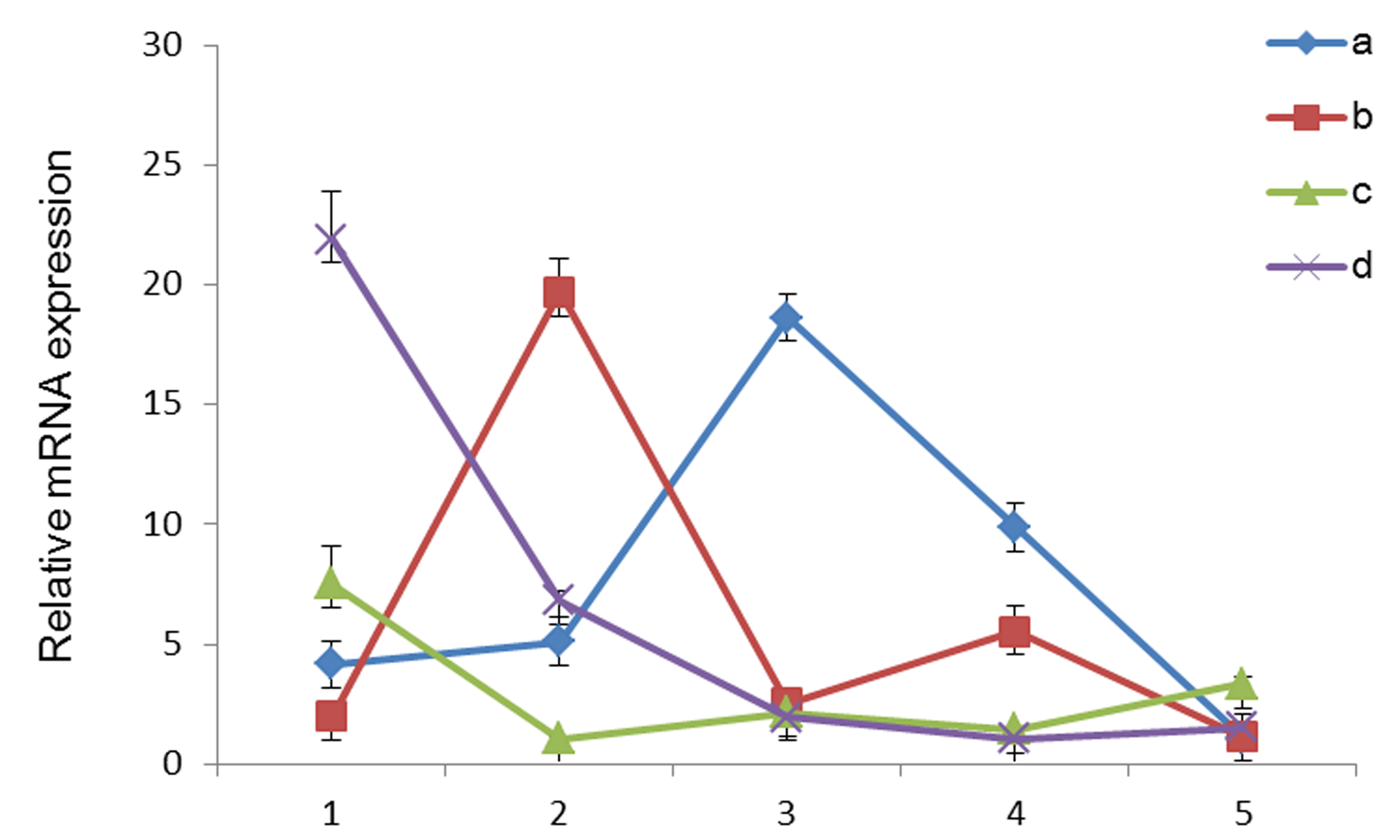

Supplement: Figure S4 — Temporal mRNA expression profiles of Tianfu goat MYOZ2 gene during different muscle development. Note: The samples 1-5 represent the 1st day, 75th day, 150th day, 225th day and 300th day, respectively; the samples a-d represent cardiac muscle, leg muscle, abdomial muscle and longissimus dorsi muscle, respectively. Bars represent the mean ± SE (n = 5). (TIF) [file pone.0082550.s004.tif]

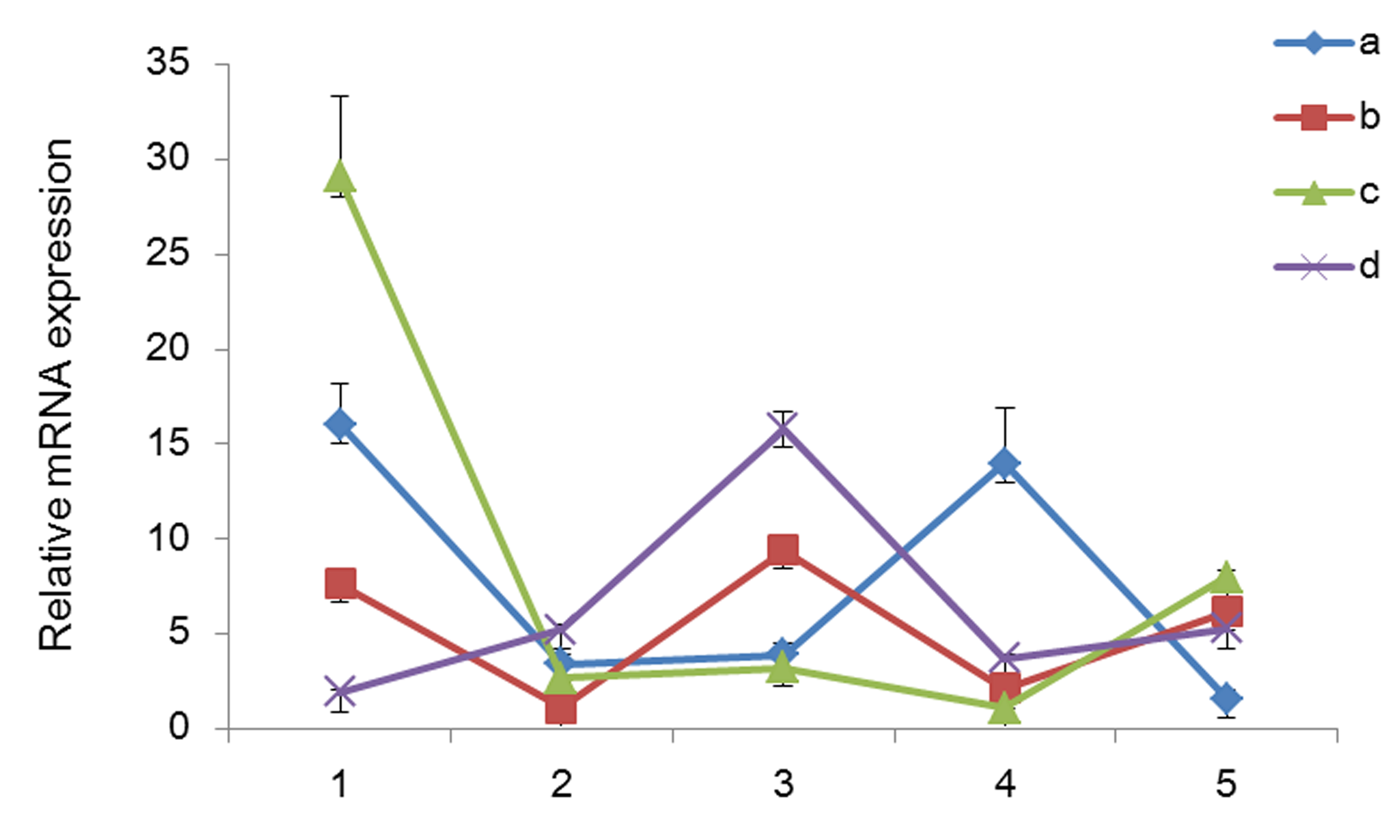

Supplement: Figure S5 — Temporal mRNA expression profiles of Tianfu goat MYOZ3 gene during different muscle development. Note: The samples 1-5 represent the 1th day, 75th day, 150th day, 225th day and 300th day, respectively; the samples a-d represent cardiac muscle, leg muscle, abdomial muscle and longissimus dorsi muscle, respectively. Bars represent the mean ± SE (n = 5). (TIF) [file pone.0082550.s005.tif]

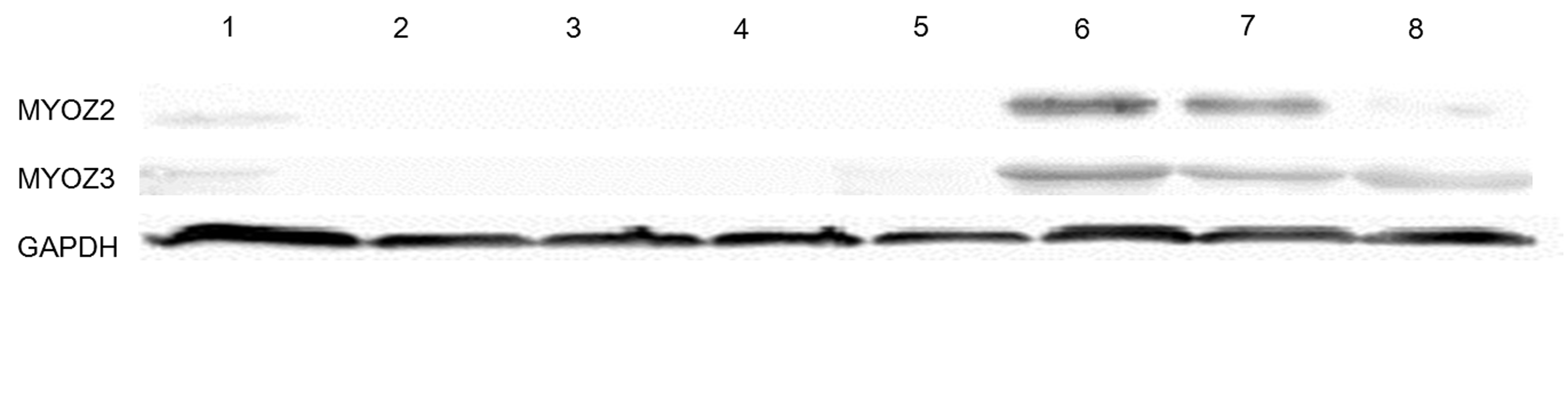

Supplement: Figure S6 — Western blotting of Tianfu goat MYOZ2 and MYOZ3 protein levels in eight tissues. Note: The samples 1-8 represent heart, liver, spleen, lung, kidney, leg muscle, abdomial muscle and longissimus dorsi muscle, respectively (n = 5). (TIF) [file pone.0082550.s006.tif]

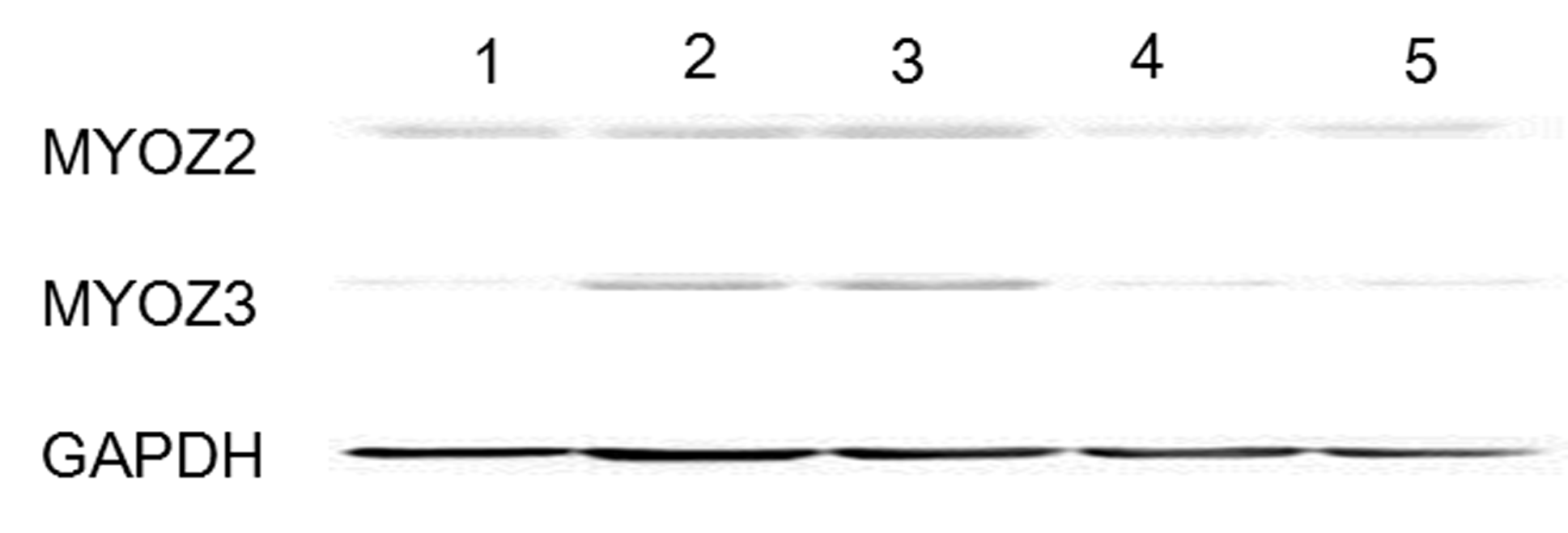

Supplement: Figure S7 — Western blotting of Tianfu goat MYOZ2 and MYOZ3 protein levels during longissimus dorsi muscle development. Note: The samples 1-5 represent the 1st day, 75th day, 150th day, 225th day and 300th day, respectively (n = 5). (TIF) [file pone.0082550.s007.tif]

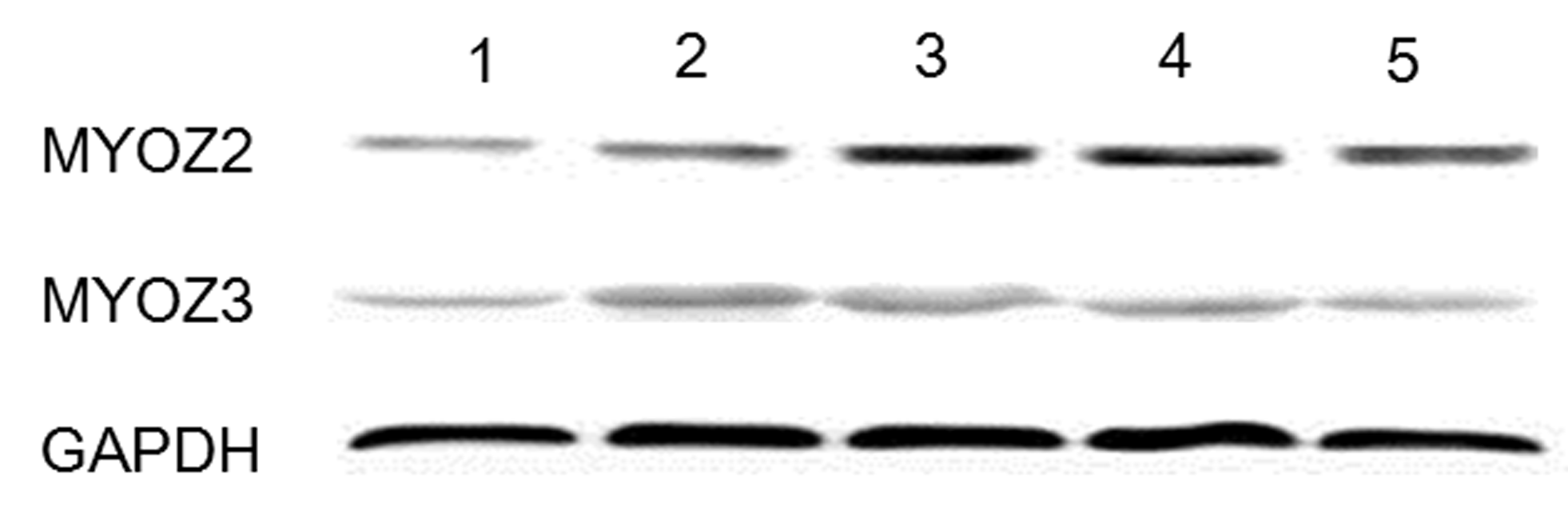

Supplement: Figure S8 — Western blotting of Tianfu goat MYOZ2 and MYOZ3 protein levels during soleus muscle development. Note: The samples 1-5 represent the 1st day, 75th day, 150th day, 225th day and 300th day, respectively (n = 5). (TIF) [file pone.0082550.s008.tif]
